# Sensors for Distributed Monitoring

**DOI:** 10.3390/s24196429

**Published:** 2024-10-04

**Authors:** Francesco Adamo, Nicola Giaquinto, Maurizio Spadavecchia

**Affiliations:** Department of Electrical and Information Engineering, Politecnico di Bari, 70126 Bari, Italy; francesco.adamo@poliba.it (F.A.); maurizio.spadavecchia@poliba.it (M.S.)

This Special Issue focuses on recent advances and applications in distributed monitoring technologies, which have progressively gained in popularity due to the growing demand for continuous measurements of large structures or areas, such as cultivated fields, pipelines, tunnels, viaducts, and so on. The output of this kind of systems is a distribution of the sensed physical quantity (e.g., temperature, strain, moisture) over time along the entire structure, or the automated detection and location of anomalous values of the monitored quantity at any point in the structure.

As is widely recognized, there are essentially two main approaches to distributed monitoring: the first consists of realizing distributed sensors, using cable-like elements (e.g., optical fibers) sensitive along their entire length; the second is based on distributed sensor networks, where several discrete sensor nodes, with wired or wireless communication, obtain the desired measurements in given positions and report them to a remote collecting and processing unit. This Special Issue is addressed to both types of distributed monitoring.

A total of thirteen papers were submitted to this Special Issue. After a careful peer review process, ten of these were accepted for publication, divided into eight research papers and two review papers. Below, we provide a brief overview of all published papers, pointing out their contributions in proposing innovative solutions in the field of distributed monitoring.

The study proposed in [1] describes an original approach for assessing road pavement health using automated data collection and processing technology. A “signal on graph” (SoG) model is introduced to enhance the detection of pavement distress. Additionally, a nonlinear Bayesian estimator is developed to improve the accuracy of distress metrics. This methodology was tested on data acquired from Kazakhstan roads, showing effectiveness in correcting errors and improving road failure detection. The used Bayesian estimator also helps identify unreliable measurements, allowing for more efficient road maintenance planning.

In [2], the development of two integrated sensors for monitoring the structural health of concrete beams is discussed. The first sensor, a diffused sensing element, detects changes in the dielectric properties of concrete along the entire beam, enabling continuous monitoring from curing through the beam’s life cycle. The second sensor, a split ring resonator network, provides precise, localized measurements of permittivity and water presence, crucial for preventing deterioration in structures exposed to water, such as dams and bridges. Additionally, these sensors can detect and localize cracks within the concrete.

In [3], the use of distributed acoustic sensing (DAS) for dynamic strain measurements of the ground is considered; these measurements are valuable for engineering applications like natural hazard monitoring and soil–structure interaction studies. The study examines whether DAS measurements align with theoretical expectations and experimental data from geophones. The results show that under specific conditions, DAS and geophone measurements are consistent in phase and amplitude. The study also discusses the implications of DAS strain measurements, including differences in directionality and magnitude, and introduces methods to improve measurement accuracy and spatial alignment.

In [4], the authors present the development and evaluation of a novel solid-phase bath (SoPhaB) for Raman backscatter-based fiber-optic distributed sensing (FODS), designed for precise temperature measurements. Unlike traditional liquid-phase water baths, the SoPhaB employs ultrafine copper, providing better accuracy and precision in geoscientific and industrial applications. The SoPhaB tightly encloses the fiber-optic cable, ensuring uniform temperature distribution within ±0.04 K, and incorporates integrated reference thermometers for calibration. The paper thoroughly details the advantages of this method, such as thermoelectric control, portability, and its effectiveness in extreme environments, offering enhanced calibration accuracy and reduced uncertainty compared to traditional techniques.

In [5], the development of a cost-effective system for monitoring the attitude and position of large structures, such as bridges and offshore platforms, using micro-electromechanical systems (MEMS) as an inertial measurement unit (IMU) is discussed. This system aims to be a more compact and affordable alternative to traditional fiber optic sensors, which are high-performing but expensive, bulky, and heavy. The proposed solution combines MEMS sensors with real-time kinematic (RTK) GPS and a real-time Kalman filter, achieving accuracy comparable to traditional methods. Tests show high reliability, with attitude estimates having standard deviations as low as 0.04° in static conditions and only slight deviations compared to fiber optic sensors in real-world simulations.

In [6], the development of a convolutional neural network (CNN) designed to detect and characterize impedance discontinuities in cables by analyzing time-domain reflectometry signals is described. The developed CNN identifies discontinuity points, classifies their types, and estimates their positions and magnitudes. Testing on both simulated and real signals showed high accuracy, with the method correctly identifying all discontinuity points in experimental tests involving capacitive faults and estimating their positions and magnitudes with minimal error.

In [7], a new technique for enhancing the sensitivity of mechanical water meters to detect small leaks, which traditional meters struggle to identify, is discussed. The technique involves an electronic add-on device that captures digital images of the meter’s mechanical register and uses image processing to detect very small water flows, even below the meter’s usual starting flow rate. Experimental tests on multijet and piston water meters demonstrated the method’s effectiveness, detecting leaks as small as 1.0–10.0 L/h for the multijet meter and 0.25–1.0 L/h for the piston meter.

Paper [8] investigates the use of distributed optical fiber sensors based on Rayleigh backscattering for monitoring strains in reinforced concrete elements under long-term external loading. The study focuses on robust fiber optic cables with steel and polymeric cladding, tested on large concrete beams over 96 days of sustained or cyclic loading. The sensors provided highly detailed strain measurements, offering new insights into the structural behavior of reinforced concrete. The sensors were accurate and stable, effectively monitoring both short-term load changes and long-term effects like creep. Comparisons with digital image correlation confirmed the accuracy of the measurements, suggesting that these sensors have strong potential for the long-term monitoring of reinforced concrete structures.

In [9], a review of advancements in using LiDAR (light detection and ranging) technology for structural health monitoring of civil infrastructure is presented. Traditional methods, such as on-site visual inspections, are highlighted as costly, time-consuming, and subjective. LiDAR, through mobile (MLS) and terrestrial (TLS) laser scanning, offers detailed geometric data for detecting structural issues like cracks, deformations, and defects over time. The review covers various applications, including monitoring bridges, roads, tunnels, and historical structures. The paper also discusses the current limitations of LiDAR technology and suggests future research directions to enhance its effectiveness in structural health monitoring.

Finally, paper [10] reviews recent advancements in road condition monitoring (RCM) from 2017 to 2022, focusing on innovative systems for evaluating pavement distress. Leveraging computer vision, data mining, and high computing power, these systems utilize next-generation sensors and AI-based methodologies to assess, classify, and localize pavement issues. The review covers both contact and noncontact measurement technologies, including smartphones, drones, and various sensors, such as RGB cameras, thermal cameras, lasers, and ground-penetrating radar (GPR). It highlights the contributions and limitations of current methods, discusses the role of smart sensors and data platforms, and identifies challenges and future research directions in AI technologies for RCM.

In conclusion, the ten papers reported in this Special Issue provide excellent examples of recent research in the area of distributed monitoring applications. We believe that readers will find the Special Issue interesting and inspiring, and that its contents will stimulate new developments in the field.

Thanks are due to all the authors for the time and effort they devoted to the preparation of their valuable works, as well as to all the highly qualified reviewers for their commitment to ensuring the quality of this Special Issue.

We would also like to thank the editorial team at MDPI for their precious and continuous support, without whom realizing this Special Issue would not have been possible.
